# Isolation and Characterization of Clinical *Listeria monocytogenes* in Beijing, China, 2014–2016

**DOI:** 10.3389/fmicb.2019.00981

**Published:** 2019-05-08

**Authors:** Xiaoai Zhang, Yanlin Niu, Yuzhu Liu, Zheng Lu, Di Wang, Xia Cui, Qian Chen, Xiaochen Ma

**Affiliations:** ^1^Beijing Center for Disease Prevention and Control, Institute for Nutrition and Food Hygiene, Beijing, China; ^2^Research Centre for Preventive Medicine of Beijing, Beijing, China

**Keywords:** *Listeria monocytogenes*, human, MLST, PFGE, antimicrobial susceptibility, China

## Abstract

*Listeria monocytogenes* is an important foodborne pathogen with a significant impact on public health worldwide. A great number of outbreaks caused by *L. monocytogenes* has been reported, especially in the United States, and European countries. However, listeriosis has not yet been included in notifiable disease in China, and thus information on this infection has been scarce among the Chinese population. In this study, we described a 3-year surveillance of listeriosis in Beijing, China. Fifty-six *L. monocytogenes* strains isolated from 49 clinical infectious cases (27 pregnancy-associated infections and 22 non-pregnancy-associated infections) were analyzed by serotyping, pulsed field gel electrophoresis (PFGE), multilocus sequence typing (MLST), and antimicrobial susceptibility testing between 2014 and 2016 in Beijing. The predominant serogroups were 1/2a,3a and 1/2b,3b,7 which accounted for 92% of the overall isolates. Four strains were serogroup 4b,4d,4e, isolated from patients with pregnancy-associated infections. Based on PFGE, these isolates were divided into 32 pulsotypes (PTs) and 3 clusters associated with serogroups. Ten PTs were represented by more than one isolate with PT09 containing the most number of isolates. MLST differentiated the isolates into 18 STs, without new ST designated. The three most common STs were ST8 (18.4%), ST5 (16.3%), and ST87 (12.2%), accounting for 46.9% of the isolates. STs prevalent in other parts of the world were also present in China such as ST1, ST2, ST5, ST8, and ST9 which caused maternal fetal infections or outbreaks. However, the STs and serogroup distribution of clinical *L. monocytogenes* in Beijing, China was different from those in other countries. Strains of ST1 and ST2 were isolated from patients with pregnancy-associated infection, whereas none of ST155 isolates caused pregnancy-associated cases. Surveillance of molecular characterization will provide important information for prevention of listeriosis. This study also enhances our understanding of genetic diversity of clinical *L. monocytogenes* in China.

## Introduction

*Listeria monocytogenes* is an important foodborne pathogen with a significant impact on public health worldwide (Schlech, 2000; de Noordhout et al., 2014). It has the potential to cause human diseases ranging from self-limited gastroenteritis and spontaneous abortion in pregnant women to severe invasive infections (sepsis or meningitis) in immuno-compromised patients or older patients (Okutani et al., 2004; Guevara et al., 2009; McCollum et al., 2013; Wang et al., 2013).

Although *L. monocytogenes* is an uncommon human pathogen, it has a disproportionate share of the food borne disease burden. A previous study reported 1600 human cases of listeriosis annually in the United States, of which 260 resulted in death (Crim et al., 2014). In the European Union, a total of 1763 confirmed human cases of listeriosis (notification rate of 0.44 cases per 100,000 population) were reported with a fatality rate of 15.6% in 2013 (European Food Safety Authority [EFSA], 2015). In China, 253 invasive listeriosis cases were reported in 19 provinces from 2011 to 2016, with a fatality rate of 25.7% (Li et al., 2018). More importantly, the incidence is still increasing worldwide, despite antibiotic treatments (Goulet et al., 2008). A large listeriosis outbreak occurred in Canada in 1981 and provided the first evidence for transmission of listeriosis by foodborne *L. monocytogenes* (Schlech et al., 1983; Evans et al., 1985). Since then, a series of outbreaks caused by *L. monocytogenes* have been reported, especially in the United States (Fleming et al., 1985; Linnan et al., 1988; Dalton et al., 1997; Gottlieb et al., 2006; Centers for Disease Control and Prevention, 2008; Chen et al., 2017) and European countries (Bula et al., 1995; Ericsson et al., 1997; Goulet et al., 1998; Aureli et al., 2000; Lyytikainen et al., 2000; Althaus et al., 2017; Amato et al., 2017; Dahl et al., 2017; Kleta et al., 2017). However, listeriosis has not yet been regulated as a notifiable disease in China and therefore information on this infection has been largely scarce among the Chinese population.

*Listeria* contains multiple species. It has been subtyped using different methods, including serotyping, pulsed-field gel electrophoresis (PFGE), multilocus sequence typing (MLST), multi-virulence-locus sequence typing (MVLST), and restriction fragment length polymorphism (RFLP). *L. monocytogenes* can be divided into 13 serotypes, of which three serotypes (serotype 1/2a, 1/2b, and 4b) are believed to cause the majority of clinical cases (Gilot et al., 1996). In all of these methods, PFGE provides higher discrimination power than serotyping, making it an important tool in source tracking and outbreak investigation (Graves and Swaminathan, 2001; Centers for Disease Control and Prevention, 2008; Chen et al., 2017; Dahl et al., 2017; Kleta et al., 2017). MLST, based on the analysis of seven housekeeping genes, has been proved as a powerful tool in molecular epidemiological studies and population structure studies of *L. monocytogenes* (Salcedo et al., 2003; Jadhav et al., 2012). However, only a few studies have approached the molecular characterization of clinical *L. monocytogenes* in China (Lv et al., 2014; Huang et al., 2015; Wang Y. et al., 2015; Zhang et al., 2016). These studies suggested that listeriosis in China was caused by heterogeneous strains. MLST types (STs) prevalence in other parts of the world were also found in China (Lv et al., 2014; Huang et al., 2015; Wang Y. et al., 2015; Zhang et al., 2016). The objective of the present study was to determine the epidemiological characteristics of listeriosis cases, as well as the characteristics of clinical *L*. *monocytogenes* isolates in Beijing, China.

## Materials and Methods

### Collection of the Clinical *L. monocytogenes* Isolates

The isolates analyzed in this study were collected from the Survey Project of Human Listeriosis in Beijing. This study was carried out in accordance with the recommendations of Manual of Foodborne Disease Surveillance, China issued by the National Center for Food safety Risk Assessment. The protocol was approved by the ethics committee of Beijing Center for Disease Prevention and Control (Beijing CDC). In this project, 12, 12, and 21 hospitals were covered in 2014, 2015, and 2016, respectively. All the suspected clinical cases of listeriosis were included in the survey. Samples were collected and used to isolate *L. monocytogenes*. We defined invasive listeriosis as isolation of *L. monocytogenes* strains from a normally sterile site or from products of conception (Li et al., 2018). All the *L. monocytogenes* isolates identified by clinical microbiology laboratories were sent to the lab in Beijing CDC.

A total of 56 isolates were isolated from 49 human patients who had a severe illness with serious suspicion of *L. monocytogenes* infection between 2014 and 2016. All the isolates were firstly identified using VITEK 2-compact system (bioMérieux, Lyons, France) or matrix-assisted laser desorption/ionization time of flight (MALDI-TOF) mass spectrometry (Bruker, Leipzig, Germany); and were further checked by PCR targeting *hly* fragments specific to *L. monocytogenes* (Xu et al., 2009).

### Serotyping

All the strains were serotyped using multiplex PCR, which was based on the amplification of the following target genes: *prs*, *lmo0737*, *lmo1118*, *ORF2110*, and *ORF2819* described by Doumith et al. (2004).

**Table 1:** The information of patients and isolates analyzed in this study.

Age	Number of isolates	Gender of patient			Whether or not pregnancy-associated infection#		The outcome of fetus or neonate		
		**F**	**M**	**UN**	**Y**	**N**	**D**	**S**	**UN**
Newborns and fetus	14	8	3	3	14	0	5	6	3
≤20	7	4	3	0	0	7	0	0	0
21∼40	24	22	2	0	20	4	13	6	1
>40	11	6	5	0	0	11	0	0	0
Total	56	40	13	3	34	22	18	12	4

*#Pregnancy-associated infections comprise listeriosis in neonates in the first month of life and maternal-fetal infections. F, female; M, male; Y, yes; N, not; S, survival; D, death; UN, unknown.*

### Antimicrobial Susceptibility Testing

Antimicrobial susceptibility testing of the *L. monocytogenes* isolates was performed using broth dilution method. The minimum inhibitory concentrations (MICs) of following 13 antimicrobials were tested: ampicillin (AMP), oxacillin (OXA), vancomycin (VAN), clindamycin (CLI), tetracycline (TET), daptomycin (DAP), erythromycin (ERY), chloramphenicol (CHL), ciprofloxacin (CIP), trimethoprim-sulfamethoxazole (SXT), gentamicin (GEN), penicillin (PEN), and cefoxitin (FOX) (Xingbai, Shanghai, China). Since resistance criteria for AMP, PEN, and SXT of *L. monocytogenes* exists in the clinical and laboratory standard institute (CLSI) international guidelines, and resistance criteria for ERY exists in European Committee on Antimicrobial Susceptibility Testing (EUCAST) international guidelines, the MICs of AMP, PEN, SXT were interpreted using CLSI international guidelines, and the MIC of ERY was interpreted according to EUCAST international guidelines. No resistance criteria exists for the other 11 antimicrobials of *L. monocytogenes*, so susceptibility to other tested antimicrobials was interpreted by those recommended for *Staphylococcus* spp. ATCC29213 was used as the reference strain.

### Pulsed-Field Gel Electrophoresis (PFGE)

Pulsed-field gel electrophoresis of the strains was processed in accordance with the PulseNet International protocol^1^. Slices of *L. monocytogenes* agarose plugs were digested using 50 U of *Asc*I and *Apa*I (Takara, Dalian, China) per slice for 3 h at 37°C; electrophoresis was performed using a CHEF-DRIII system (Bio-Rad Laboratories, Hercules, CA, United States); electrophoresis was conducted with a switch time of 4–40 s for 19 h; and images were captured using a Gel Doc 2000 system (Bio-Rad) then converted to TIFF files. *Salmonella enterica* serovar Braenderup strain H9812 restricted with *Xba*I was used for molecular weight determinations in all PFGE gels. The TIFF files were analyzed using BioNumerics software (version 7.6 Applied Maths, Kortrijk, Belgium). Clustering was performed using the unweighted pair group method with arithmetic mean (UPGMA).

### Multilocus Sequence Typing (MLST)

Multilocus sequence typing was performed on all the 56 isolates by amplification and sequencing of internal fragments of seven housekeeping genes (*abcZ*, *bglA*, *cat, dapE, dat*, *ldh*, and *lhkA*) (Salcedo et al., 2003). Sequencing was performed on an ABI 3770 automatic sequencer. The alleles and sequences types (STs) were determined by comparison with the allelic profiles for *L. monocytogenes* in MLST database^2^.

BioNumerics software was used to create a cluster tree and conduct minimum spanning trees (MST) based on the allelic profiles. In MST, a clonal complex (CC) was formed by STs with six of seven MLST alleles in common and at least two STs; the founder ST was defined as the ST with the highest number of single-locus variants (SLVs); single genotypes that did not correspond to any clone groups were defined as singletons. Types were represented by circles; size of a circle indicated the number of strains of this particular type. Lineages of isolates were assigned as Wiedmann et al. (1997) described.

## Results

### Origin of the Strains

The origins of the strains were summarized in Table 1. Fifty-six strains were isolated from the 49 cases. Among them, 27 were pregnancy-associated infections, in which all mothers were cured; ten neonates were survived, whereas thirteen fetuses died in the womb or after birth. No data were available for the four fetuses. Among the other 22 non-pregnancy-associated patients, 11 (50%) were males. The median age of patients with non-pregnancy-associated infections was 42 years, five patients were >60 years, and five patients were ≤3 years.

### Characterization of the Isolates From the Same Patient

**FIGURE 1:**
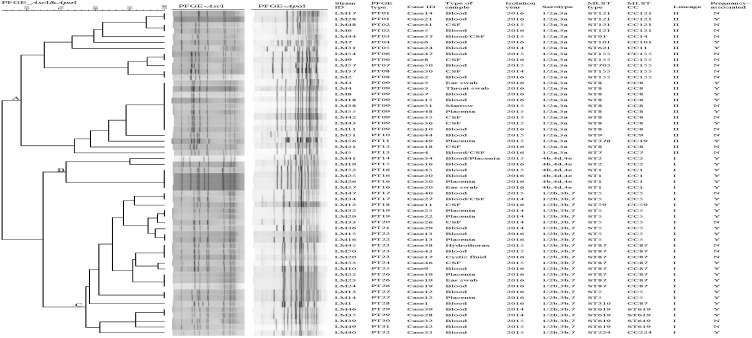
Relationships of the isolates based on PFGE. Forty-nine clinical *Listeria monocytogenes* strains were analyzed by PFGE using *Asc*I and *Apa*I. The corresponding data, including the name of the strain (Strain ID), the name of the case (Case ID), PFGE types, the type of sample, isolation date, serotype, MLST type (ST), MLST clonal complexes (CC), lineage, and pregnancy-associated were shown alongside the dendrogram to the right.

There were five groups of isolates from five patients (Figure 1). The isolates from the same patient had the same serogroup, antimicrobial susceptibilities, PFGE type, and ST. Therefore, only one isolate from each patient was used for the subsequent analysis.

### Lineages and Serogroups

Lineage was determined based on the MLST data. We found that 25 strains belonged to lineage I and 24 strains to lineage II. Almost half of the strains belonged to serogroup 1/2a,3a (*n* = 24,49%) followed by serogroup 1/2b,3b,7 (*n* = 21,43%), serogroup 4b,4d,4e (*n* = 4, 8%). Strains of serogroup 1/2b,3b,7 and serogroup 4b,4d,4e belonged to lineage I, whereas strains of serogroup 1/2a,3a belonged to lineage II. All 4 strains of serogroup 4b,4d,4e were isolated from patients with pregnancy-associated infections. More pregnancy-associated cases were caused by lineage I than lineage II strains (72 vs. 37.5%, respectively).

### PFGE

Pulsed-field gel electrophoresis analysis of the comprised *Asc*I and *Apa*I divided 49 isolates into 32 pulsotypes (PT01-P32) (Figure 1). PT09 accounted for 16.3% (8/49) of isolates, followed by PT23 (3 isolates, 6.12%). Eight PTs (25%) were represented by two isolates. Twenty-two PTs (44.9%) were represented by only one single isolate. A UPGMA dendrogram was constructed for the 32 PTs based on presence or absence of bands. PTs were divided into 3 clusters, respectively (cluster A, B, and C) (Figure 1), corresponding to the three serogroups identified.

### MLST

**FIGURE 2:**
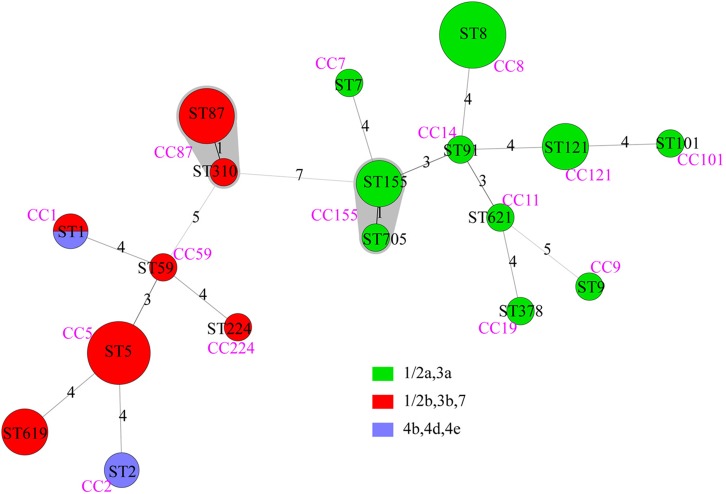
The minimum spanning tree of STs of 49 clinical *L. monocytogenes* strains isolated from Beijing, China. Each circle represents one sequence type. Gray zones surrounding groups of STs represent CC. The size of the circle is proportional to the number of the isolates and the color within the cycles represents the serotypes of the isolates. Links between circles are represented according to the number of allelic mismatches between STs.

Forty-nine isolates were divided into 18 STs, with no new ST designated. Lineage I included 8 STs of serogroups “1/2b,3b,7” and “4b,4d,4e”; lineage II included 10 STs of serogroups “1/2a,3a” (Figure 2). The most common STs were ST8 (9 isolates, 18.4%, “1/2a,3a”), ST5 (8 isolates, 16.3%, “1/2b,3b,7”), ST87 (6 isolates, 12.2%,“1/2b,3b,7”), followed by ST121 (4 isolates, 8.2%, “1/2a,3a”), ST155 (4 isolates, 8.2%, “1/2a,3a”), and ST619 (4 isolates, 8.2%, “1/2b,3b,7”). Other 12 STs contained one to two isolates, respectively (Figure 2). Among the 18 STs, 17 of them could be assigned clonal complexes and one (ST619) was a singleton based on querying the MLST database (Figure 2). Strains of ST1 and ST2 were isolated from patients with pregnancy-associated infection, whereas none of the ST155 isolates caused pregnancy-associated cases. Five of the nine ST8 strains were isolated from patients with pregnancy-associated infection among which three fetuses died. Six of the eight ST5 strains were isolated from patients with pregnancy-associated infection. Fetuses of pregnancy-associated cases caused by ST5 isolates died while those caused by ST87 isolates survived (except 1 lost to follow-up).

### Comparison of Isolates Obtained From This Study to Other Cities of China

**FIGURE 3:**
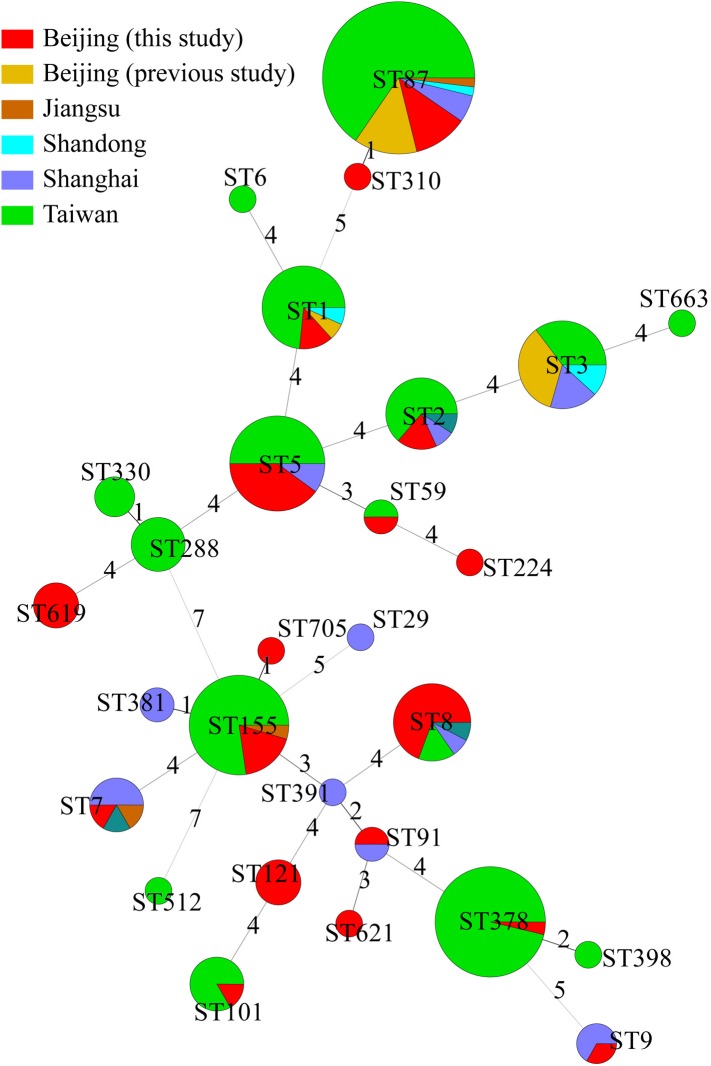
Genetic relationships of the 225 Chinese isolates. A minimum spanning tree was constructed based on STs of 49 isolates from this study and 176 isolates from other studies in China. Each circle represents a sequence type. The size of the circle is proportional to the number of the isolates, and the sources of the isolates were colored as shown in figure. Links between circles are represented according to the number of allelic mismatches between STs.

The 49 isolates in this study were compared with 176 other isolates from cases of clinical illness in China reported in previous studies (Lv et al., 2014; Huang et al., 2015; Wang Y. et al., 2015; Zhang et al., 2016). These samples were isolated from Beijing (*n* = 14), Jiangsu (*n* = 3), Shandong (*n* = 4), Shanghai (*n* = 20), Shanxi (*n* = 3), and Taiwan (*n* = 132). Together, these 225 isolates were divided into 28 STs. Among them, 13 ST were found in different regions. There were no obvious regional characteristics of STs (Figure 3).

### Comparison of Isolates With Other Countries

**FIGURE 4:**
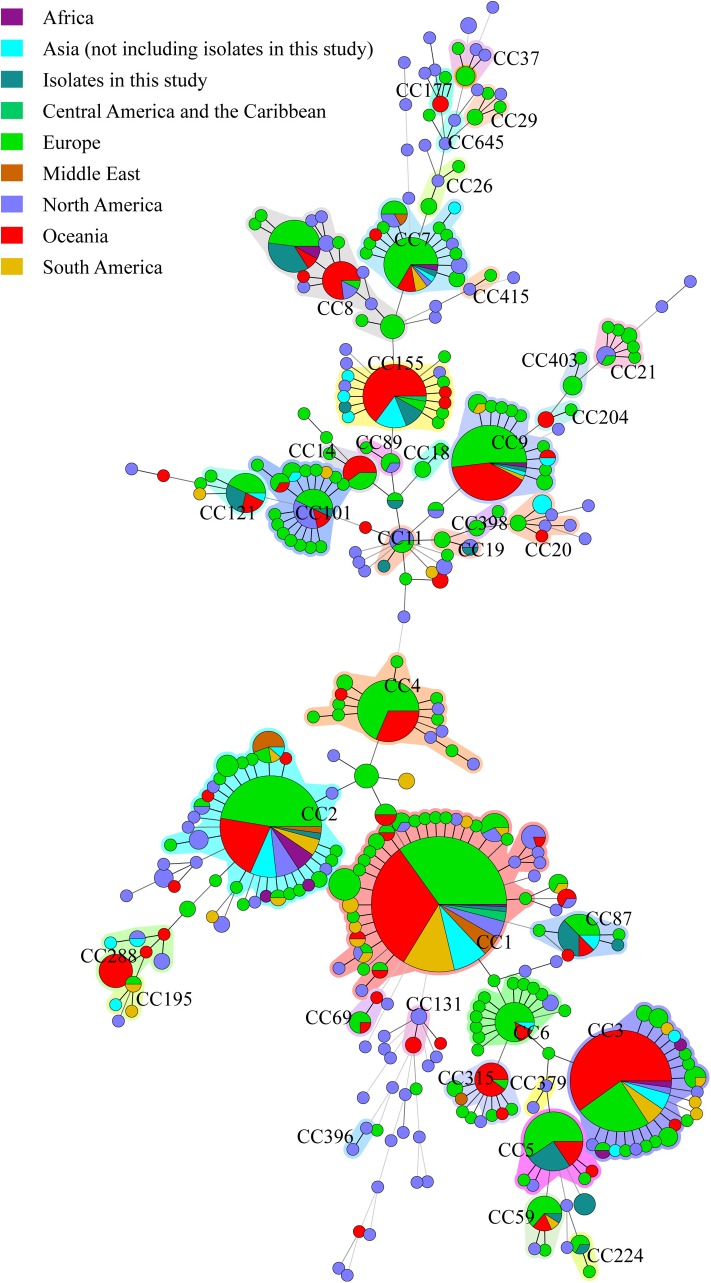
Genetic relationships of the 56 clinical isolates in Beijing and 1094 global isolates from *L. monocytogenes* MLST database. A minimum spanning tree was constructed based on CCs from this study and MLST database. The size of the circle is proportional to the number of the isolates, and the sources of the isolates were colored as shown in figure. The shadow zones in different color represent different clonal complexes.

The 49 isolates were compared with clinical *L. monocytogenes* isolates from rest of the world (Figure 4). A total of 1094 human *L. monocytogenes*, screened out from *L. monocytogenes* MLST database see text footnote 2 in October 08, 2017, were included in the analysis. The 1143 human isolates were divided into 38 CCs and 87 singletons. The most globally prevalent CC in the database were CC1 (224), CC2 (133), CC3 (118), CC9 (65), CC4 (49), CC7 (46), CC8 (45), CC155 (41), and CC101 (32). All isolates obtained in this study, excepting ST619, were co-clustered with foreign isolates. The 15 CCs detected in this study were also found in at least two other countries (Supplementary Table S1).

### Antimicrobial Susceptibilities of the Isolates

Antimicrobial susceptibility testing was conducted for the 49 *L.monocytogenes* isolates. Details were listed in Table 2. The highest resistance rate was observed for FOX, which reached 100%, followed by DAP (93.9%), OXA (85.7%), and CIP (36.7%). In contrast, three other antimicrobials (TET, 6.1%; PEN, 4.1%; GEN, 2.0%) had resistance rates lower than 10.0%. All the isolates were susceptible to AMP, VAN, CLI, ERY, CHL, and SXT. Among all the 49 isolates, 79.6% (39/49) were co-resistant at least to OXA, DAP, and FOX; 28.6% (14/49) were co-resistant at least to OXA, DAP, CIP, and FOX. Seven isolates (ST5, ST8, ST7, ST155, and ST705) were sensitive to OXA, three isolates (ST5, ST378) were sensitive to DAP, while seventeen isolates (ST1, ST5, ST310, ST619, ST121, ST91, ST8, ST7, and ST155) were resistant to CIP, three isolates (ST155, ST705) were resistant to TET, two isolates (ST101, ST8) were resistant to PEN, one isolates (ST8) was resistant to GEN (Table 2).

## Discussion

In China, a few studies have reported the prevalence of *L. monocytogenes* in food (Chen et al., 2009; Wang et al., 2012; Wu et al., 2016). However, the descriptions of clinical *L. monocytogenes* are very limited. In this study, we described the characteristics of molecular subtyping and antimicrobial susceptibilities of clinical *L. monocytogenes* strains in Beijing, the capital city of China. The clinical strains analyzed in this study were isolated from a systematic investigation, which provided a unique opportunity to investigate the characterization of this important pathogen in China.

Our findings, for the first time, revealed the predominant serogroups and STs of clinical *L. monocytogenes* in Beijing, China. The results showed that there were some differences in clinical *L. monocytogenes* serogroups distribution between Beijing and other countries. Among the 49 isolates in this study, the predominant serogroups were 1/2a,3a (49%) and 1/2b,3b (45%). The serogroup compositions were in agreement with that of previous study, showing 28 *L. monocytogenes* isolates collected from patients from four cities/provinces in China (Wang Y. et al., 2015). The predominant serogroups were also 1/2a,3a and 1/2b,3b in strains isolated from ready-to-eat foods, raw foods and edible mushrooms in China (Chen et al., 2015, 2018a; Wu et al., 2015, 2016). While, the predominant serogroups of clinical *L. monocytogenes* strains in our study were different from those from the other countries such as United States, Australia, Brazil, Italy and Portugal (Mammina et al., 2009; Bueno et al., 2010; Centers for Disease Control and Prevention, 2014; Magalhaes et al., 2014; Almeida et al., 2017; Jennison et al., 2017). In United States, serotype 4b (57%) was the most commonly identified serotype of *L monocytogenes*, followed by serotype 1/2a (26%) and serotype 1/2b (13%) (Centers for Disease Control and Prevention, 2014). In Australia, serogroup 4b,4d,4e was the predominant group, accounting for 56.6% of clinical isolates (Jennison et al., 2017). In Italy, 1/2a and 4b were the predominant serotypes, representing 46.3 and 42.6% of human isolates from sporadic cases (Mammina et al., 2009).

Previous molecular epidemiological studies have detected unreported human outbreaks of listeriosis, for example, Ariza-Miguel et al. (2015) identified an epidemiological connection among strains via analysis of the genomic relationships among isolates through PFGE and MLST subtyping. In our study, ten PFGE clusters possessed strains that were isolated from 2 or more different cases. The isolates with same PFGE patterns had no obvious link as the isolates were obtained from different time and places, with no evidence of epidemiological association. It should be noted that isolates from the case38 and case43 patients, which were identified in the same hospital for 6 days apart, shared an indistinguishable PT, suggesting a possible related source. On the other hand, five groups of isolates from same patients in this study had the same serogroups, molecular subtypes and antimicrobial susceptibility profiles, indicating dissemination of *L. monocytogenes* in body. In case21, we isolated a *L. monocytogenes* strain from a beef sandwich in the market, where the pregnant patient always bought foods. This isolate had the same PFGE pattern with the strain LM28, indicating that beef sandwich may be the source of infection (Wang and Chen, 2016). In case13, the PFGE pattern of strains LM15, and LM16 were consistent with a strain isolated from the patient’s home environment swab, indicating possibility of cross contamination (unpublished data).

Most of STs identified in this study were largely distributed across world countries and regions, showing that “everything is everywhere” paradigm of *L. monocytogenes* clones (Chenal-Francisque et al., 2011). Many STs identified in this study have been associated with outbreaks in other countries, such as ST1 caused outbreaks in France in 1989 and in Sweden in 1995, ST2 caused an outbreak in Italy in 1997, and ST5 strains caused outbreaks in Canada in 1996 as well as in United States in 2011 (Ericsson et al., 1997; Aureli et al., 2000; Lomonaco et al., 2013). In addition, CC3, CC4, CC8, CC88, CC87, CC398, and CC403 were associated with outbreaks in MLST database.

**Table 2:** The resistance rates of 49 *L. monocytogenes* isolates.

Antimicrobial	Susceptibility			Resistance			Resistance rate (%)
	Number of strains	PT	ST	Number of strains	PT	ST	
Cefoxitin (FOX)	0	–	–	49	01∼32	1, 2, 87, 5, 310, 59, 224, 619, 121, 91, 101, 621, 8, 9, 7, 378, 155, 705	100
Daptomycin (DAP)	3	17, 22	5, 378	46	01∼21, 23∼32	1, 2, 87, 5, 310, 59, 224, 619, 121, 91, 101, 621, 8, 9, 7, 155, 705	93.9
Oxacillin (OXA)	7	06, 07, 08, 09, 13, 22	5, 8, 7, 155, 705	42	01∼05, 10∼12, 14∼21, 23∼32	1, 2, 87, 5, 310, 59, 224, 619, 121, 91, 101, 621, 8, 9, 378, 155	85.7
Ciprofloxacin (CIP)	31	01∼12, 14∼18, 20, 21, 23∼17, 29∼32	1, 2, 87, 5, 59, 224, 619, 121, 101, 621, 8, 9, 378, 155, 705	18	01∼03, 06, 08, 09, 13, 16, 19, 22, 27, 28, 31	1, 5, 310, 619, 121, 91, 8, 7, 155	36.7
Tetracycline (TET)	46	01∼06, 09∼32	1, 2, 87, 5, 310, 59, 224, 619, 121, 91, 101, 621, 8, 9, 7, 378, 155	3	07, 08	155, 705	6.1
Penicillin (PEN)	47	01∼03, 05∼08, 10∼32	1, 2, 87, 5, 310, 59, 224, 619, 121, 91, 621, 8, 9, 7, 378, 155, 705	2	04, 09	101, 8	4.1
Gentamicin (GEN)	48	01∼32	1, 2, 87, 5, 310, 59, 224, 619, 121, 91, 101, 621, 8, 9, 7, 378, 155, 705	1	09	8	2.0
Ampicillin (AMP)	49	01∼32	1, 2, 87, 5, 310, 59, 224, 619, 121, 91, 101, 621, 8, 9, 7, 378, 155, 705	0	–	–	0
Vancomycin (VAN)	49	01∼32	1, 2, 87, 5, 310, 59, 224, 619, 121, 91, 101, 621, 8, 9, 7, 378, 155, 705	0	–	–	0
Clindamycin (CLI)	49	01∼32	1, 2, 87, 5, 310, 59, 224, 619, 121, 91, 101, 621, 8, 9, 7, 378, 155, 705	0	–	–	0
Erythromycin (ERY)	49	01∼32	1, 2, 87, 5, 310, 59, 224, 619, 121, 91, 101, 621, 8, 9, 7, 378, 155, 705	0	–	–	0
Chloramphenicol (CHL)	49	01∼32	1, 2, 87, 5, 310, 59, 224, 619, 121, 91, 101, 621, 8, 9, 7, 378, 155, 705	0	–	–	0
Trimethoprim-sulfamethoxazole (SXT)	49	01∼32	1, 2, 87, 5, 310, 59, 224, 619, 121, 91, 101, 621, 8, 9, 7, 378, 155, 705	0	–	–	0

In our study, CC8/ST8 clone was the most common ST, which was distributed globally For instance, in Switzerland, CC8 was the most prevalent clone during 2011–2013 (Althaus et al., 2014); and in Denmark, a CC8/ST8 clone was associated with most of sporadic cases during 2004–2012 (Jensen et al., 2016). Both CC8 clones in Denmark and Canada did not lead to pregnancy-associated infections but were mainly associated with elderly patients (Knabel et al., 2012; Jensen et al., 2016), which was different from our study. In our study, CC8 caused five pregnancy-associated infections. Some studies suggested that the CC8 strains from Canada possessed a strong capacity of biofilm formation, which might support persistence within food production environments, resulting in subsequent contamination of foods (Verghese et al., 2011). In this study, eight CC8/ST8 strains had the indistinguishable pulsotypes; the fact that identical pulsotype had been found in the same period raised the possibility that contamination of food could originate from a common source.

CC5/ST5 clone was the second common ST in our study. Similarly, ST5 was the most predominate ST in ready-to-eat meat product in Nanjing, China (Wang G.Y. et al., 2015). In France, no obvious difference was observed between frequency distribution of CC5 in food samples and clinical samples (Maury et al., 2016). Although CC5 was not considered as a hypervirulent clone in the study of Maury et al. (2016) CC5 caused several outbreaks in the recent years, such as a multistate cantaloupe outbreak in US in 2011 (Lomonaco et al., 2013), a multistate ice cream outbreak in US in 2010–2015 (Centers for Disease Control and Prevention, 2015), and a stone fruit recall in US in 2014 (Jackson et al., 2015). In our study, all fetuses of pregnancy-associated cases caused by ST5 isolates died. Further studies are needed to uncover the virulence of CC5.

Interestingly, ST87 was seldomly linked to human infections in other countries. In 2008, one ST87 isolate from water was reported by Ragon et al. (2008). In 2014, Perez-Trallero et al. (2014) reported two outbreak episodes caused by ST87 strains affecting 15 people in northern Spain. In 2012, ST87, a common MLST type, had been reported from foodborne strains in China (Wang et al., 2012). In 2015, ST87 was reported to be the most frequent ST from clinical strains in Taiwan from 2000 to 2013 (Huang et al., 2015). The ST87 were also found as the most common ST in isolates from rodents in nature environment in China (Wang et al., 2017). Besides, we searched CC87 in MLST database and found that there were a CC87 strain isolated from human in Japan in 1988 see text footnote 2. Our study showed that 12.2% of the human isolates were ST87, demonstrating that ST87 has been already circulating in Beijing. It is necessary to follow the dissemination of this clone to assess its potential emergence. Comparing with ST5, fetuses of pregnancy-associated cases caused by ST87 isolates survived. More studies will be required to further assess the virulent diversity of *L. monocytogenes* in different STs.

It is widely accepted that food is the source of human *L. monocytogenes*. The comparison of the population structure of the clinical strains in our study with that from foods, revealed that all STs, except ST621, were also reported in foodborne isolates in China (Wang et al., 2012; Wang Y. et al., 2015; unpublished data). However, there was a notable difference in prevalence of STs between human isolates and foodborne isolates. The most common types of isolates from food sources were ST9, ST8 and ST87 (Wang et al., 2012). ST87 and ST8 were predominant in fresh aquatic products and edible mushroom products in China (Chen et al., 2018a,b). Consistently, ST8 and ST87 were the most predominant STs in human isolates in our study. ST9 (72%) was the most common ST in strains isolated from 356 raw pork samples, 2104 raw pork retail environment swabs and 329 insects in China (Wang et al., 2017). MLST analysis of isolates from France showed a higher prevalence of ST9 and ST121 isolates among food sources when compared with those clinical original (Maury et al., 2016).

Some studies reported that reduced susceptibility of *L. monocytogenes* to many antibiotics in various countries and increases in the frequency of multidrug-resistant strains (Pesavento et al., 2010; Korsak et al., 2012). In our study, the prevalence of some common antibiotic resistance among *L. monocytogenes* isolated from patients in China was relatively low. PEN alone or with gentamycin is the prioritized antibiotic for treating human listeriosis. Sulfamethoxazole and trimethoprim is used in patients having PEN anaphylaxis. ERY is used in treating pregnant women. Meanwhile, VAN is used in treating *L. monocytogenes* bacteremia and endocarditis (Janakiraman, 2008). In this study, no resistance to SXT, ERY, VAN, AMP, CLI, and CHL was found, and there was a relatively low resistance rate to PEN (4.1%) and GEN (2.0%). Strains isolated from a Spanish hospital were found to be sensitive to AMP and ERY (Ariza-Miguel et al., 2015), which was consistent with our study. However, most *L. monocytogenes* isolated from food and food processing environments in China and other countries were resistant to PEN (Obaidat et al., 2015; Lotfollahi et al., 2017; Chen et al., 2018b), which may result from different resistance criteria use. *L. monocytogenes* is intrinsically resistant to FOX which had a resistance rate of 100% in our study. High resistance to OXA, as reported in previous studies (Khen et al., 2015; Wang et al., 2017), may be intrinsic. In the study, no correlation was found between antimicrobial susceptibility patterns and other characteristics, such as serogroups, PFGE, and MLST types. Further studies are required to survey the antibiotic susceptibility of clinical *L. monocytogenes* and to explore the potential molecular mechanisms of antibiotic resistance in *L. monocytogenes.*

## Conclusion

In summary, this study observes that more than half of listeria cases were pregnancy-associated infections in Beijing, and as such more attention should be paid to pregnant patients in future studies on this infection. The serogroup and ST distribution of clinical *L. monocytogenes* in Beijing was different from many other countries. This study enhances our understanding of genetic diversity of clinical *L. monocytogenes* in China. Continuous surveillance for this pathogen in clinical patients is necessary in China.

## Author Contributions

YN and XM were involved in the collection of isolates and collected the clinical data. YL, ZL, DW, and XC performed the molecular subtyping and antibiotic susceptibility tests. XZ performed the data analysis. XZ and QC designed the study, drafted, and revised this manuscript.

## Conflict of Interest Statement

The authors declare that the research was conducted in the absence of any commercial or financial relationships that could be construed as a potential conflict of interest.

## Footnotes

1 http://www.cdc.gov/pulsenet/pathogens/index.html

2 http://bigsdb.pasteur.fr/listeria/listeria.html
