# Supplementary material for: Isolation and Characterization of Clinical Listeria monocytogenes in Beijing, China, 2014–2016
Source: Front Microbiol. 2019 May 8;10:981. doi: 10.3389/fmicb.2019.00981 (PMC6517826; doi:10.3389/fmicb.2019.00981)
Supplement: Supplementary file 1 [file Table_1.DOCX]

Supplementary Table S1 The information of CCs of this study in the *L. monocytogenes* MLST database^*^

| CC (ST) | No. of isolates in this study | No. of isolates in the database | No. of countries that contains this CC in the database | Regional distribution of isolates in the database |
| --- | --- | --- | --- | --- |
| CC1 (ST1) | 4 | 222 | 28 | Africa, Asia, Central America and the Caribbean, Europe, Middle East, North America, Oceania, South America |
| CC101 (ST101) | 1 | 32 | 10 | Asia, Europe, North America, Oceania, South America |
| CC11 (ST621) | 1 | 7 | 4 | Europe, North America |
| CC121 (ST121) | 4 | 11 | 6 | Asia, Europe, Oceania |
| CC14 (ST91) | 1 | 16 | 6 | Europe, North America, Oceania |
| CC155 (ST155, ST705) | 5 | 41 | 9 | Asia, Central America and the Caribbean, Europe, North America, Oceania |
| CC19 (ST378) | 1 | 3 | 2 | Europe, North America |
| CC2 (ST2) | 2 | 131 | 24 | Africa, Asia, Europe, Middle East, North America, Oceania, South America |
| CC224 (ST224) | 1 | 3 | 3 | Europe |
| CC5 (ST5) | 10 | 27 | 4 | Europe, North America, Oceania |
| CC59 (ST59) | 1 | 12 | 9 | Europe, North America, Oceania, South America |
| CC7 (ST7) | 1 | 44 | 14 | Africa, Asia, Europe, Middle East, North America, Oceania, South America |
| CC8 (ST8) | 10 | 44 | 9 | Africa, Europe, North America, Oceania |
| CC87(ST87, ST310) | 9 | 12 | 4 | Asia, Europe, Oceania |
| CC9 (ST9) | 1 | 64 | 14 | Africa, Asia, Europe,Oceania,South America |
| ST619 | 4 | 0 | 0 | - |

* The database website is http://bigsdb.pasteur.fr/listeria/listeria.html. The querying and statistics were done in October 8, 2017.
